# Circadian Preferences and Coping Styles to Stressful Life Events in Depression Patients

**DOI:** 10.1192/j.eurpsy.2024.1094

**Published:** 2024-08-27

**Authors:** P. Güzel Özdemir, T. Ülkevan, M. Işık, E. Sütçü

**Affiliations:** Van Yuzuncu Yil University, Van, Türkiye

## Abstract

**Introduction:**

Depressive disorder is a common public health problem that significantly impairs quality of life and has a high risk of mortality and morbidity.

**Objectives:**

The aim of this study was to investigate circadian rhythm differences, stressful life events and coping styles in patients with depression.

**Methods:**

The study involved 100 participants, including 50 patients with depression and 50 healthy controls, recruited from the psychiatric clinic of one-university hospital. The participants completed a sociodemographic information form, Beck Depression Inventory (BDI), Life Events Checklist (LEC-5), Coping Inventory for Stressful Situations-Short Form (CISS-21) and Morningness-Eveningness Questionnaire (MEQ).

**Results:**

The mean age of the patients with depression was 31.88±10.6 years, and the control group was 29.84±8.02 years. There were no significant relationships between the variables including gender and some other sociodemographic characteristics except education level. There were significant differences between the depression and control groups in terms of coping styles for stressful life events. Emotional coping was significantly higher in patients with depression compared to the control group, whereas task-oriented coping was significantly lower than the control group (p<0.05). The majority of both depression and the control group consisted of intermediate type. Natural disasters, severe suffering, and other stressful events or experiences were more frequent stressful life events in the depression group. Task-oriented coping scores and emotional coping scores showed significant discrimination with sensitivity and specificity values.

**Conclusions:**

Recognizing stressful life events and the coping strategies used to deal with them is important for identifying future mental problems such as depression and developing treatment and follow-up plans. Longitudinal studies are needed to fully understand how the reporting of mature and dissociative coping methods interacts with depression in recovery from traumatic events.

**Disclosure of Interest:**

None Declared

